# On the Kinetics of Degradation Reaction Determined Post Accelerated Weathering of Polyolefin Plastic Waste Blends

**DOI:** 10.3390/ijerph16030395

**Published:** 2019-01-30

**Authors:** S.M. Al-Salem, M.H. Behbehani, H.J. Karam, S.F. Al-Rowaih, F.M. Asiri

**Affiliations:** Environment & Life Sciences Research Centre, Kuwait Institute for Scientific Research, P.O. Box: 24885, Safat 13109, Kuwait; mbahbaha@kisr.edu.kw (M.H.B.); hjkaram@kisr.edu.kw (H.J.K.); shruwaih@kisr.edu.kw (S.F.A.-R.); fasiri@kisr.edu.kw (F.M.A.)

**Keywords:** plastic, polyolefin, degradation, solid waste, thermolysis, kinetics

## Abstract

Polyolefin (PO) polymers constitute the majority of consumer plastic commodities. The reliance on such materials make it near imposable to avoid touching one in any given day. Therefore, the accumulation of plastic solid waste (PSW) in developed and developing societies alike requires immediate attention to manage and valorize this type of waste. In this work, PSW originating from real life sources and virgin linear low-density polyethylene (LLDPE) films were compounded in a mechanical recycling effort. The recycled blends constituted up to 100% (by weight) of the waste material. Accelerated weathering (aging) was conducted on the blends, reaching threshold limit of exposure to study the major changes occurring on the recycled blends. Thermogravimetry and differential scanning calorimetry (DSC) were used to determine their characteristics and applicability for future recycling using thermo-chemical treatment (TCT) methods. Analytical solution methods following the international committee of thermal analysis and calorimetry (ICTAC) were followed in conducting the measurements and kinetic calculations alike. A novel analytical mathematical solution model is also introduced to determine both the pre-exponential factor (A_o_) and apparent activation energy (*E_a_*) of the degradation reaction. The model proved to be a more accurate analysis tool, and the work in whole enabled the determination of future plans for using such waste components as a feedstock to thermal units.

## 1. Introduction

### 1.1. Dependency on Plastics and Environmental Pollution

Ever since their appearance back in the 1930s, plastics have dominated the consumer market with a continuous increase in demand and production capacity ever since. The global production of plastics reached 335 million tonnes in 2016, representing over 600% increase since 1975 [1]. The majority of virgin plastic resin is used to cover the overwhelming demand of the packaging sector. These materials encompass a verity of polyolefin (PO) polymers, including familiar household grade names such as linear low density polyethylene (LLDPE), low density polyethylene (LDPE), high density polyethylene (HDPE), and polypropylene (PP). These materials encompass essential products making the attempt to avoid touching them near impossible on a daily basis. In addition, the majority of these products are converted to plastic films designed for single use and disposal [2]. This contributes immensely to the accumulation of plastic solid waste (PSW) in both terrestrial and marine environments. This burdensome phenomena on the environment is associated with adverse effects including migration of heavy metals and chemicals, open dump explosions, and aquifer poisoning [3]. The majority of the chemicals that may be released during the various stages of the plastic’s life cycle (e.g., from production to handling and disposal) are classed as hazardous according to European Union (EU) classification and labelling regulation which is based on the United Nations Globally Harmonized System (UNGHS) [4]. This is due to the fact that the majority of these plastics are made of monomers derived from non-renewable fossil fuels [5].

The generation of solid waste (SW), and in particular PSW, is associated with societal level of comfort, gross domestic product (GDP) index, population, and urbanization [6]. These factors lead to alarming estimates of high PSW per capita generation and an overwhelming mass of plastics carried by waterways reaching to 4.2 million tonnes per day [7]. The Organization for Economic Co-operation and Development (OECD) countries have the highest waste generation rate with an average of 2.2 kg per capita per day, retaining 44% of global estimates [8]. On an individual country basis, small and developing island nations are noted to be the highest producers of waste in the world. The State of Kuwait is a small developing country with a recent waste generation estimate of 5.7 kg per capita per day, ranking first among the world’s countries [9]. PSW makes over 16% of the municipal solid waste (MSW) generated in Kuwait [10]. This corresponds to over 200,000 million tonnes of PSW generated on an annual basis estimated to be over 13% of household SW [11,12]. These estimates surpass industrial nation generation rates commonly associated with PSW accumulation. In both the USA and the UK, PSW make up 12.1% and 9.9% of the total MSW load, respectively [13,14].

Locally, the State of Kuwait is in need of good practices and national strategies that can take advantage of the accumulated SW. This will ultimately rid the environment from stressors such as toxic chemicals migration by leaching, harmful emissions from additives, bacteria growth, and greenhouse gases (GHGs) emissions [15,16,17]. All of the previously stated cause concerns that have been reported in the past are present within this region of the world [3]. The country also lacks appropriate infrastructure that is capable of dealing with SW in general. The sole disposal method of MSW in Kuwait is unsanitary landfilling. Currently, there are 18 landfill sites in Kuwait, of which 15 sites are closed [18]. These landfills act as disposal sites, rather than engineered landfills with energy recovery systems capable of processing waste from dry or wet materials recovery facilities (MRF). Major concerns are also increasing regarding landfilling in Kuwait where environmental issues and public health are put in question due to its excessive use.

### 1.2. Recovery and Valorization of Plastics

It is a well-established fact that plastics’ presence in landfills cause adverse environmental effects. Past studies showed that the disposal of PSW in landfills seizes an accumulated 100% equivalent of carbon from the atmosphere due to loss of potential energy, products and materials [19]. In addition, space requirements for landfill operation presents a major issue, promoting recycling routes of PSW and energy recovery. Appropriate management of PSW reduces health concerns associated with plastic pollution and exposure. Recent studies show that di-(2-ethylhexyl)phthalate (DEHP), the common plasticizer additive used in polyvinyl chloride (PVC) manufacturing, can be correlated to a ‘cocktail effect’ of health concerns including increased waist circumference and insulin resistance [20]. On the other hand, bisphenol-A (BPA) is associated with destructive health effects including early sexual maturation, decreased male fertility and aggressive behavior [19]. BPA is a main ingredient in the majority of adhesives and epoxy plastics which can be detected in plastic bottles.

Discarded plastics can be valorized via four routes, in an ascending order of preference, termed as the hierarchy for plastic waste management [21]. These routes encompass several techniques that render the PSW article as a viable product. These routes start with re-extrusion methods, where the waste material is re-introduced into the production line of the virgin material. The second order of preference is given to mechanical recycling, where the PSW is sorted and washed before utilizing physical processes to compound blends comparable to the virgin plastic product. Finally, PSW is treated via chemical and thermal means to produce feedstock materials, monomers, and energy. The last two processes are termed tertiary and quaternary methods encompassing thermolysis techniques [5]. These methods include pyrolysis and gasification with the aim of chemicals and fuel recovery. Nonetheless, mechanical recycling is the preferred method of choice when it comes to recycling PSW, especially of in film forms. It presents various advantages that makes it a lucrative option, such as ease of operation and handling (since the equipment utilized is mostly similar to virgin plastic compounding), reduction of waste accumulation and the recovery of easily marketable products.

On the other hand, it is a must to maintain the integrity of a material recycled via mechanical means after introducing it to the heating cycles and loops in the recycling schemes. The recycled product must have comparable properties to virgin plastic products on the market, in order to, deem the recycling process successful. Mechanically recycled plastics are prone to rheological changes in the structure of their constituting polymer [22]. In addition, thermal degradation occurs to the polymeric chains due to photo-oxidation effects from oxygen diffusion during re-processing stages [12]. The PSW feedstock is typically used in a single type polymer batch after sorting and materials are preferred to be freshly discarded with their properties and integrity still intact.

Investigating the effect of compounding PSW as a material of choice with virgin polymers is a practice that possess a gap in research and development (R&D). Typically, reinforcing agents and additives are added to virgin polymers in order to increase their integrity and durability. Such fillers and agents include wood fibers, short and fiber glass, and compatibilizer [23,24]. Such a practice is typically noted for PO polymers with the aim of using them for film applications (e.g., covers and bags, etc.). In doing so, the effect of adding PSW to the polymeric matrix is still in need of research, especially if the aim is to produce durable products that can withstand harsh climatic and weathering conditions (similar to those encountered in the Middle East region). An increase in PO consumption of 20 million tons between the years 2005 and 2015 was noted for the Middle East (ME) region alone. This is a reflection of other Asian regions including China, India and the Fareast, where the per capita consumption is higher than the European one (38 kg per person) by a considerable margin. Such an increase also plays a major factor in the increase of the total SW load of ME countries. The use of PSW as a filler to virgin polymers can be of great added value both economically and environmentally. This is in addition to the development of recycling techniques in blending and compounding the PSW with virgin materials as fillers.

Weathering of polymers is a practice that has been popularized for its ability to determine both changes in properties and the integrity of plastic products. It is a standardized test method that can take place in controlled environments (e.g., accelerated aging) or outdoor conditions (e.g., natural weathering.). Table 1 summarizes the major findings of the main studies conducted in polymer weathering in recent years. Studies that investigate degradation reaction changes occurring to the plastic material due to weathering and exposure to environmental conditions, are very scant in scientific literature [25,26]. These types of research works can determine the applicability of the plastic material for recycling via thermal and thermo-chemical treatment (TCT) such as pyrolysis (e.g., treatment of SW under inert atmospheres). Investigating the degradation kinetics can also provide an insight on the materials changes under pyrolytic conditions. Therefore, this work has been initiated to characterize various blends of PSW that are of plastic film in origin compounded with virgin commercial extrusion film grade plastic (i.e., LLDPE). The work presented here results from experimental and mathematical modelling efforts that aim at investigating the degradation reaction kinetics of mechanically recycled blends after exposure to various weathering cycles. The exposure of the materials was conducted in-line with internationally recognized experimental protocols to reflect climatic conditions that waste can be exposed too. These materials can be further treated using various thermal units, which require a full understanding of the degradation reaction kinetics. The materials chosen are commercially available and are typically used for various application. This makes the work presented here of major relevance to societies aiming at recycling their PSW via thermolysis and TCT techniques. To the best of the authors’ knowledge, there is no study that resembles the work carried out in this communication.

## 2. Materials and Methods

### 2.1. Materials Acquirement and Samples Compounding/Recycling

Discarded real life PSW in the form of films was secured from a local dealer in the amount of 200 kg. The PSW was used as the material of choice as it represents reclaimed SW of municipal origin in Kuwait originating from commercial and residential sources. The material was randomly sampled following the procedures depicted in ASTM D 1898-68 [32]. The constituting elements of the PSW were determined by melting point identification using a TA instrument Q-series model differential scanning calorimeter (DSC); subjecting a 9 ± 0.1 mg sample in the crucible equilibrated at 40 °C for 5 min. Readers are referred to Al-Salem et al. [33] for full description of the procedure as it falls out of the scope of this article. The material was segregated and cleaned for further processing [33]. The PSW contained the following polymer as constituting elements (by wt%): LLDPE (46%), LDPE (51%), HDPE (1%); and PP (2%) [34]. The PSW was milled to 3 mm flakes using a Tecnova cutting mill and pelletized to 3 mm pellets using a Tecnova single screw extruder (L/D = 30) at 40 bars pressure and 70 rpm. The cooling water temperature was maintained between 16 and 17 °C [12,33,34]. The recycling procedure aimed at dry blending (without the addition of additives) LLDPE with the PSW in different weight ratios. This particular resin was chosen as it is the most common plastic type used for film applications namely in the packaging sector similar to the reclaimed PSW. LLDPE (EFDC-7050 extrusion grade) was graciously supplied by EQUATE Petrochemical Company (DOW Chemicals, Midland, MI, USA) and used in this work as white translucent pellets with a density of 0.918 g·cm^−3^ and a melt flow index (MFI) of 2 g·10 min^−1^. The supplying company did not provide details of the additives for confidentiality reasons. The plastic blends were extruded and blown using a single screw extruder (Tecnova, L/D = 30, 45 bars and 85 rpm) and a film blowing machine (Kung Hsing monolayer, Kung Hsing Plastic Machinery, Chia-Yi Hsien, Taiwan) with a water-cooling temperature maintained between 16 °C and 17 °C using a die head temperature of 175 °C [12]. Figure S1 in the supplementary materials file shows the undertaken recycling stages. The virgin LLDPE to PSW ratios (in weight) considered in this study were (virgin/waste) 50/50, 25/75 and 0/100. These codes will be used throughout this communication to refer to the various formulated blends. The extruded sheets of 100 m thickness were cut using a standard cutting die (Ray Ran UK cutting press) to produce 20 × 1 cm films for further characterization. The thickness was chosen as an average value for majority indoor and outdoor applications of plastic film products. The samples appeared consistent with no visual fractures or crazing and were comparable to each other by touch (Figure S2). All samples were stored in laboratory conditions at 23 °C/50% relative humidity (RH) and in the dark between sample formulation and testing. Readers are referred to Al-Salem et al. [12] for more details.

### 2.2. Accelerated Weathering

Accelerated (artificial) indoor weathering (ageing) tests are commonly used for studying materials integrity. This study aims at comparing the impact of weathering before exposure and exposure to threshold limit which was established previously for the testes specimens as 11 days of continuous accelerated weathering exposure, equivalent to 264 days of natural outdoor environment [12]. The exposure to accelerated weathering will determine the ability of recycling the tested materials using thermolysis. Films of the different formulations were exposed to weathering tests in accordance with ASTM D 4329 [35]. The conducted tests were accomplished laboratory conditions after mounting the samples on the weathering machine racks facing the UV lamps with no empty spaces in the panels (Figure S3). This is in order to maintain a uniform repeatable test conditions. Cycle A procedure was used for general applications durability testing, i.e., 8 h of UV exposure at 60 °C followed by 4 h of condensation at 50 °C [29]. At the end of each continuous weathering test, the chamber was cooled to room temperature and trays were set to rest on a flat surface for a minimum of 24 h. Samples were laid to rest for a minimum of 72 h before characterization [33]. A minimum of four replicates were exposed to the different exposure duration in the QUV machine chamber. Ultraviolet (UV) lamps irradiance was also selected according to ASTM D 4329 [35], and the lamp type was set to be 0.68 W·m^−2^ (irradiance) for normal operation. The irradiance sensor was calibrated every 400 h of lamp operation during the UV cycle under normal test temperature. The equipment used was cleaned every 800 h to remove scale deposits resulting from water evaporation during the condensation cycles (Figure S3).

### 2.3. Differential Scanning Calorimetry (DSC)

A Perkin Elmer (Model Jade) coupled with PYRIS analysis software (PerkinElmer Inc., Waltham, MA, USA) was used to test control and exposed samples using a 5 ± 0.1 mg samples taken from the middle section of the film samples. Aluminium oxide (Al_2_O_3_) crucibles made were used for both samples and reference materials. Crystallinity measurements were determined using scans of the first and second heating cycle between 50 °C and 230 °C based on the peak area of the heat flow curve between 60 °C and 130 °C, with a nitrogen (N_2_) gas flowrate of 20 mL·min^−1^ and a heating rate of 10 °C·min^−1^. Cooling rate was set at 15 °C·min^−1^ in similar conditions [36,37]. The first heating cycle was used in the crystallinity analysis as it will show the inherited effects of thermal histories and weathering effect on the specimens [38]. The crystallinity was estimated as the ratio of the melting enthalpy of the specimen’s measurement to a 100% crystalline polyethylene (e.g., 293.6 J·g^−1^) [38].

### 2.4. Thermogravimetric Analysis (TGA)

Thermal degradation of virgin/waste blend samples was investigated using a Shimadzu TGA-50 thermobalance (Kyoto, Japan) equipped with a data acquisition/analysis software (TA Instrument, New Castle, DE, USA) set to record the data every second under five heating rates (*β*) (i.e., 5, 10, 15, 20, and 25 °C·min^−1^). A constant flow of pure (99.99%) dry nitrogen with a flow rate of 50 mL·min^−1^ was maintained throughout the experiments. The measurements were conducted using 5 ± 0.1 mg samples from room temperature to 550 °C showing high repeatability in accordance with the International Confederation for Thermal Analysis and Calorimetry (ICTAC) recommendations previously published in Vyazovkin et al. [39,40] for non-isothermal (dynamic) thermogravimetry [25,26,41].

### 2.5. Kinetics of Degradation Analysis and Mathematical Modeling Framework

The degradation reaction was studied in this work following the TGA analysis with the aim of comparing the apparent activation energy (*E_a_*) estimated for each film sample under pyrolytic conditions. To this end, the ICTAC recommendation were followed for performing the kinetics analysis [39,40]. The material’s conversion was estimated at first and was the basis for all computations in this work. The conversion of the material (α) is typically defined, with respect to reaction time, as [25]

(1) $$\alpha =\frac{m-m_{o}}{m_{o}-m_{f}}$$

where *m* is the mass of polymeric material at a specific time of reaction (*t*), *m_o_* and *m_f_* are the initial and final mass of the polymer at the investigated temperature desired and final reaction time, respectively. The rate for degressive kinetics is typically defined according to the change in reaction rate by incorporating the first order for of the Arrhenius equation as per

(2) dαdt=Aoexp(−EaRT)f(α)

where A_o_ stands for the pre-exponential (or frequency) factor (min^−1^), *E_a_* is the apparent activation energy (kJ·mol^−1^), T is the reaction temperature at desired time (K) and f(α) is the reaction model that represents the degradation mechanism following a reaction order typically denoted as *n*. Experimentally estimated parameters are termed apparent (or empirical) in such cases to indicate possible deviation from intrinsic parameters of a certain individual step [25,26,39,40]. The Kissinger method is an integral multiple heating rate method that has been widely applied in the past [42,43]. The method relies on applying an approximation of the temperature’s integral and assuming that the *E_a_* is constant. Henceforth, the following expression can be obtained.

(3) ln(βTm2)=−EaRTm−ln[EAoR∫0∞d(α)f(α)]

where *β* is the heating rate (°C·min^−1^) and *T_m_* is the maximum degradation temperature. A plot of ln(βTm2) vs. 1/T_m_ makes it possible to determine the value of *E_a_* for each polymer or blend [26]. The isoconversional kinetics of the Flynn–Wall–Ozawa (FWO) method was also applied in this work to estimate the *E_a_* values described previously by Aboulkas et al. [43].

(4) $$ln\left(\beta \right)=ln\left[\frac{A⇄E_{a}}{g\left(\alpha \right)R}\right]-5.331-1.052\left(\frac{E_{a}}{RT}\right)$$

A linear relationship is obtained by plotting ln(*β*) vs. 1/T at an interval of 5% polymer conversion and the *E_a_* is obtained from the straight line’s slope. Readers are referred to Table S1 for a depiction of the g(α) and f(α) expression used in kinetics computation are previously described in past works [40,44,45]. The model fitting method of Criado was also used in this work to estimate the *E_a_* value. The model is defined with a Z(α) function for the solid-state reaction as per [25]

(5) Z(α)=dαdtβπ(χ)T

where $\chi =E_{a}/RT$ and π(χ) is an approximation of the temperature integral expressed with the fourth order rationale expression, i.e., P(x), of Senum and Yang. The master curves were obtained in accordance to Equation (6) with respect to the models shown in Table S1 [40,44,45].

(6) Z(α)=f(α) g(α)=dαdT (Ea/R)·exp(EaRT)P(x)

Vyazovkin et al. [40] approximated Equation (6) to yield the following term for the Z(α)

(7) $$Z\left(\alpha \right)=f\left(\alpha \right)\cdot g\left(\alpha \right)=\left(\frac{d\alpha }{dt}\right)T^{2}\left[\pi \left(\chi \right)/\beta T_{\alpha }\right]$$

Assuming that the term $\left[\pi \left(\chi \right)/\beta T_{\alpha }\right]$ has a negligible effect; hence, the values of Z(α) can be obtained by the multiplication of dα/d*t* and T^2^. Z(α) were plotted using Equation (7) following Polleto et al. [44] methodology.

In order to reflect the degradation of polymeric blends, a mathematical model was established with respect to each degradation stage in the TGA thermogram following the methodology previously discussed in [46]. Since the polymeric blends in this study are all of PO polymer origin, there was a singular step of degradation that can be distinguished in the thermograms. The model also accounts for this particular degradation behavior. To this end, the kinetic parameters were evaluated by developing an analytical solution model for the studied thermal degradation reaction of the blends. Rearranging the degressive kinetics expression previously shown in Equation (2) yields the following expression for a known time (*t*, min) with respect to the polymer fraction (*x_p_*) which can be obtained by subtracting unity from the conversion value.

(8) $$\frac{dx_{P}}{x_{p}^{n}}\;=-k\cdot dt$$

The reaction is assumed to be of *n*th order. Both sides of Equation (8) were integrated from time equals to zero (polymer fraction, *x_p_* = 1) to *t*. The real integral limits results in

(9) $$\frac{x^{-n+1}}{-n+1}=\;-k\cdot t$$

Rearranging the denominator after substituting *k* with the Arrhenius expression shown in Equation (3) results in the following expression as described previously by other authors [25,26,46].

(10) $$x=\;{\left(\left(n-1\right)Aexp(-E/RT)t+1\right)}^{-1/\left(n-1\right)}\;\mathrm{for}\;n\;\neq \;1$$

The optimization problem is given in the following mathematical manner

(11) $$\mathrm{Objective}\;\mathrm{Function}\;\left(O.F.\right)⇄\;=\;\min\overset{N}{\underset{i=1}{\sum }}\left|\frac{x_{P}{}_{\left(\exp\right)}-x_{p}{}_{\left(\mathrm{th}\right)}}{x_{p}{}_{\left(\exp\right)}}\right|.$$

Where N is the number of model solution time steps, representing the degradation stages with respect to temperature in the thermobalance of the TG. The O.F. is the summation of the expression in Equation (11). *x_p(exp)_* and *x_p(th)_* reflect the experimental and model values with respect to the solution at each given time of the degradation reaction [46]. The model solved is subject to the following constraints showcasing limitations of the mathematical formulae

(12) $$E_{a}\geq ⇄\;0$$

(13) $$t_{o}=0$$

(14) $$x_{p}(t_{o})=1$$

The first and second boundary conditions, subjects the mathematical formulae for a positive response to the solution established whilst minimizing the solutions optimized by the software. The third limiting boundary condition, subjects the polymer fraction at the beginning of the reaction to be equal to unity—i.e., 100 wt.%—with respect to the time of reaction equaling to zero [46]. The O.F. considered in this work encompasses three variables (i.e., A_o_, *E_a_* and *n*) optimized to match experimental *x_p_* as a function of t and T. The multi variable non-linear optimizations is based on a pre-set *β* against computed values [25,26,46]. The kinetics evaluation conducted in this study was previously established in our past work [25].

## 3. Results and Discussion

### 3.1. Crystallinity and Thermal Stability

PO waste can be recycled using thermolysis techniques. Past studies have shown the applicability of pyrolysis in treating virgin and waste plastics alike [5,21,25]. To the best of our knowledge, this study is the first to report the results shown for virgin and waste blends. The crystallinity estimated for the studied specimens using the DSC analysis were used to determine the changes occurring in the polymeric matrix due to the weathering. The degree of crystallinity for the unexposed 50/50, 25/75, and 0/100 samples were estimated to be 33, 30, and 28%, respectively (Table S2). The crystallinity is shown for the first heating cycle to reflect the effect of weathering on the studied specimens. This shows that the materials possess large areas of non-crystalline zones conforming with past semi-crystalline polymers results [38,47]. On the other hand, these results also indicate that the incorporation of waste into the virgin LLDPE structure can alter the degree of crystallinity (Table S2). This could be attributed to the immiscibility of both types of materials and the lack of compatibilization agents’ presence [48]. It was also noted that waste material addition increases the crystallinity to a certain degree before reducing it to a value similar to the estimated one for virgin LLDPE (25%) [49]. The architecture of the polymers chains is suspected to alter with the addition of the PSW, hence a reduction in the crystallinity. The fluctuating behavior noted with the crystallinity can also be attributed to the thermal history of the PSW. The waste material has undergone various heating loops and cycles. The recycling of the plastic material leads to the dispersion of particles. This can lead to the nucleation and size distribution within the structure of the polymer. Depending on the waste content, heterogeneous nuclei can be activated [50].

**Figure 1:**
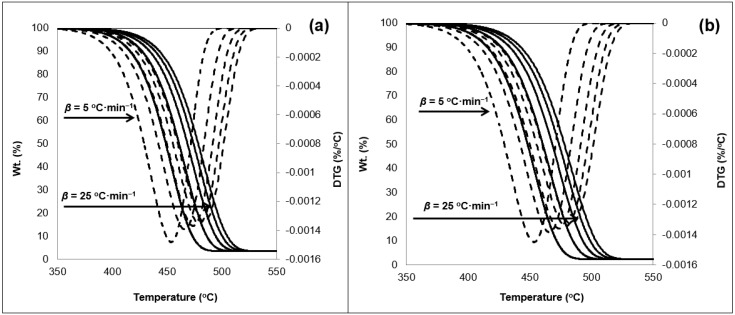
TGA thermogram showing weight loss (—) and first derivative (- - -) of the 50/50 sample (**a**) pre-exposure and (**b**) exposed to 11 days in N_2_ atmosphere with respect to heating rates (5 to 25 °C·min^−1^) in sequential order.

The TGA data of the 50/50 sample is shown in Figure 1. The thermogram indicates a singular decomposition step under a nitrogen atmosphere ensuring pyrolytic conditions. A near zero mass is noted towards the termination of the reaction. Exposure to weathering conditions reduced the remaining char resulting from the 50/50 samples tests conducted under the various *β*. A residue estimated as 3.5% remained as a solid char in the TGA crucible for the unexposed samples (Figure 1a). This is noted to be 1% higher that the exposed samples shown in Figure 1b.

**Figure 2:**
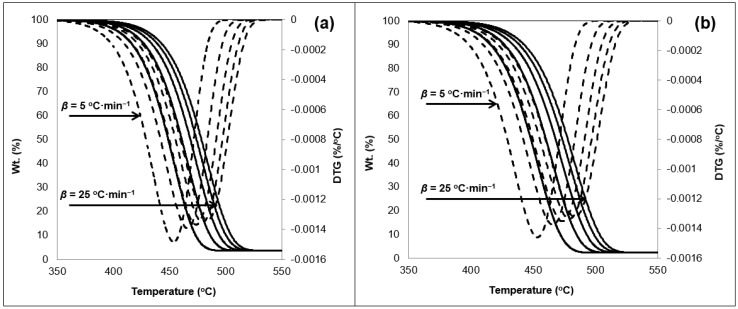
TGA thermogram showing weight loss (—) and first derivative (- - -) of the 25/75 sample (**a**) pre-exposure and (**b**) exposed to 11 days in N_2_ atmosphere with respect to heating rates (5 to 25 °C·min^−1^) in sequential order.

The exposure to weathering results in the embrittlement of the samples due to cross-linking [25]. In addition, the change in the degree of crystallinity discussed in the previous section; weakens the bonds of the polymers. This is with respect to change in amorphous region and voids reduction in the whole matrix. All of which can loosen the polymeric matrix which can lead to an increased deterioration noted with the char measured at the termination of the experiment. The 50/50 thermogram also revealed one inflection point before and after exposure to weathering, which was noted on the DTG curve. A clear shift in the thermal response was also noted as a result of the change in the *β* values (Figure 1). This conforms with past investigations on PO polymers thermal response behavior [25,41,51]. The onset temperature measured at 5 wt% loss of material, did not change as a result of the weathering test. This indicates that photo-degradation, and by association photo-oxidation, did not alter the temperature required to degrade the material. The range of the onset temperature was detected between 404 °C and 430 °C for the various *β* values considered. Readers are referred to Tables S3 and S4 for full temperature profile data of the TGA thermograms. There was no effect noticeable on both the midset temperature (50 wt% weight loss) and the maximum degradation temperature (Tables S3 and S4). Figure 2 shows the TGA thermogram obtained for the 75/25 samples.

**Figure 3:**
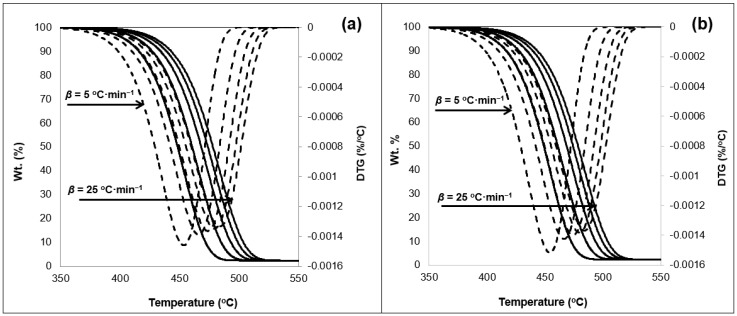
TGA thermogram showing weight loss (─) and first derivative (- - -) of the 0/100 sample (**a**) pre-exposure and (**b**) exposed to 11 days in N_2_ atmosphere with respect to heating rates (5 to 25 °C·min^−1^) in sequential order.

Residual char was also noted to reduce as a consequence of the exposure to accelerated weathering by the amount of 1.4 wt% of the original sample charge. On the other hand, the thermogram behavior did not get affected it terms of the temperature profile (see Tables S3 and S4). This supports the claim that discarded waste PSW and weathering (e.g., photo-degradation) will not impact thermolysis treatment of such materials. Figure 3 shows the TGA thermograms of the 0/100 samples, i.e., 100 wt% PSW material.

The residual char was witnessed to be at minimal levels in the case of the 0/100 samples. The char reduced from 2.4 wt% in the unexposed samples (Figure 3a) to 2.3 wt% (Figure 3b). Pyrolysis will typically result in three distinctive products, namely tars, light gases, and solid char [5]. The char is comparable to carbon black and can be utilized as such. On the other hand, the reduction of the char yield indicates the increased potential of utilizing non-condensable gases and tars which can be utilized as fuels from the PSW. The treatment of PSW minimizes the char production in the micro balance, as a result of the treatment of the exposed materials to weathering. This can also be considered as an advantage for the utilization of waste as a feedstock material in such processes. This is also supported by the fact that no transitional shifts were detected in the thermogram as a result of the weathering tests. This points towards high stability of the studied materials.

### 3.2. Model Free Kinetics Methods and Master Curve Study

**Figure 4:**
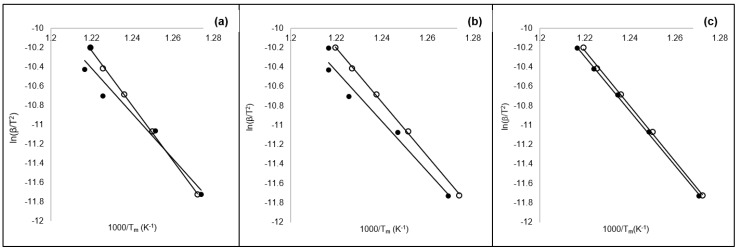
Determination of apparent activation energy (*E_a_*) using Kissinger’s isoconversion method for unexposed (○) and exposed (●) samples of the (**a**) 50/50, (**b**) 25/75, and (**c**) 0/100 blends.

The kinetic parameters, namely the *E_a_* values, were estimated before and after exposure to weathering tests. This was conducted as an indication of the required energy for deteriorating the materials in pyrolytic conditions. Figure 4 shows the Kissinger method plot for the studied materials. The dataset showed very good linearity with a standard error for *E_a_* values below 5%. A slight shift in both of the 50/50 and 25/75 datasets was noted (Figure 4a,b). This could be attributed to the two degradation stages of both the virgin and waste plastic materials. The estimated *E_a_* for the unexposed 50/50, 25/75, and 0/100 materials were 236.71 kJ·mol^−1^, 231.72 kJ·mol^−1^, and 236.71 kJ·mol^−1^, respectively. The *E_a_* values for the exposed materials were 195.34 kJ·mol^−1^, 215.48 kJ·mol^−1^, and 232.91 kJ·mol^−1^. Generally, *E_a_* values decreased with exposure to weathering showing consistency with maximum degradation temperatures among the tested materials. This reflects the fact that the single step kinetic method of Kissinger can be used to estimate the degradation kinetics of waste materials exposed to climatic conditions [26,40].

**Figure 5:**
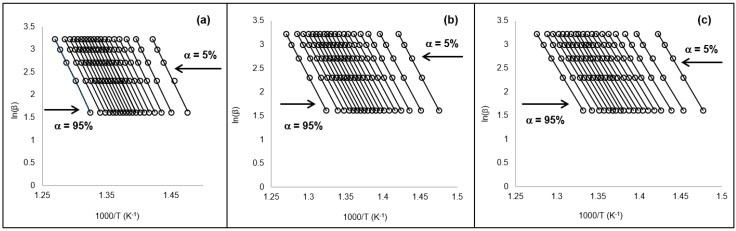
OFW method determination plot for apparent activation energy (*E_a_*) showing (**a**) 50/50, (**b**) 25/75, and (**c**) 0/100 unexposed samples. Note: conversions in sequential order from right (5%) to left (95%) with 5% intervals.

**Figure 6:**
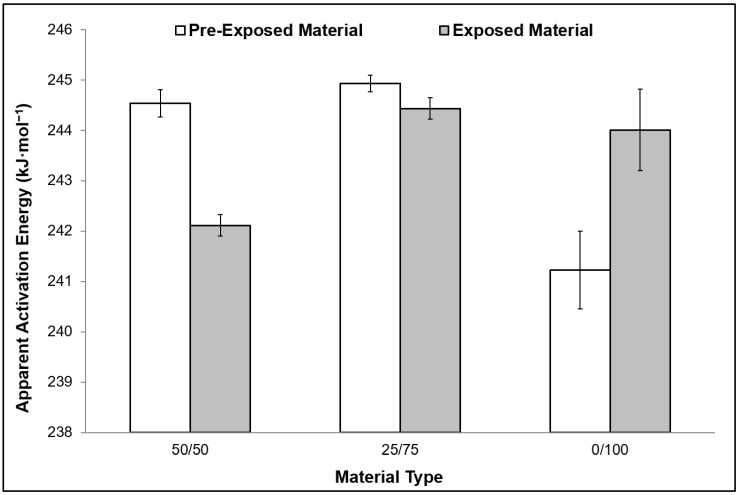
Variation in apparent activation energy between the pre-exposed and exposed materials determined using the OFW isoconversion method.

The FWO method was also used to determine the *E_a_* values (Figure 5). A consistent trend was noted among all tested materials, in-line with PO polymers analysis reported in past works [25,26,43,44]. Generally, a minor decrease in the *E_a_* values after total exposure to the threshold limit of accelerated weathering; was detected among the studied samples (Figure 6). The values also align with the isoconversion analysis values estimated in this work. The unexposed 50/50, 25/75, and 0/100 materials values of *E_a_* were estimated at 244.5 kJ·mol^−1^, 244.93 kJ·mol^−1^, and 241.22 kJ·mol^−1^, respectively. The values of the exposed materials were 242.11 kJ·mol^−1^, 244.43 kJ·mol^−1^, and 244.0 kJ·mol^−1^, respectively (Figure 6). Standard error was estimated in this work and values were less than 5%.

**Figure 7:**
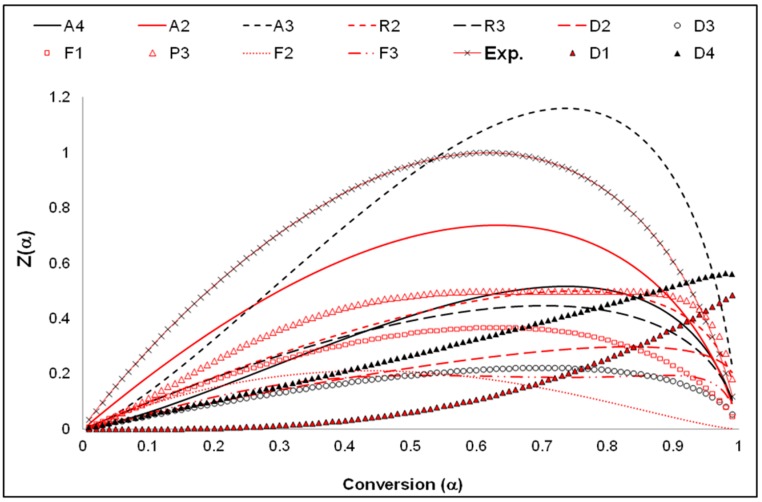
Mechanism determination master curve using Criado method for pre-weathered 50/50 samples.

The master curves obtained from the Criado model were used to determine the mechanism that represents the dataset of both the unexposed and exposed materials [40,43]. The four designated model groups depicted in Table S1 (i.e., A, R, D, and F) represent different forms of degradation mechanisms. These forms depend upon the mathematical derivation of mass and heat transfer [40]. The A_3_ model which stands for the model representing the nucleation and growth of (Avarami equation), represents the dataset obtained for unexposed and exposed materials alike. A sample of the master curves is shown in Figure 7 for the 50/50 material. This analysis is also consistent with previous PO polymers investigation on virgin grades of PE and PP [25,26]. 

**Figure 8:**
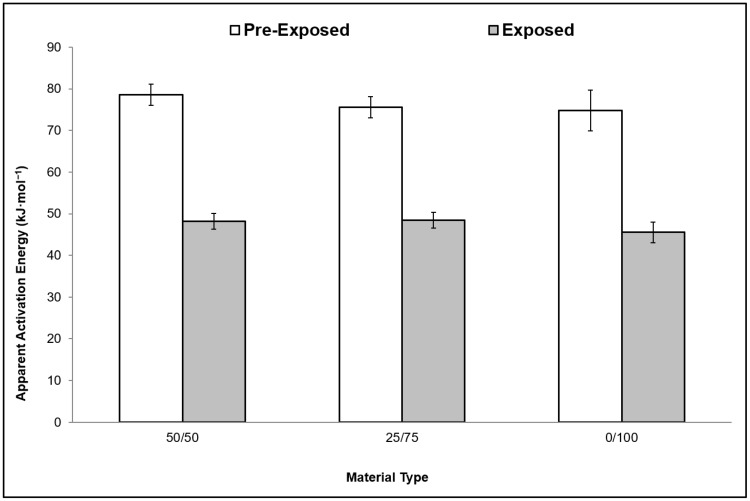
Variation in apparent activation energy between the pre-exposed and exposed materials determined using the Criado method.

The Criado method was also used to estimate the *E_a_* values of the studied materials, with the variation of the results depicted in Figure 8. A decrease in *E_a_* values was observed after exposure to accelerated weathering. The *E_a_* values were reduced by almost half their original values. The results of the Criado method take into account the temperature of the degradation, hence show lower values of *E_a_* [25]. On the other hand, the kinetics parameters in this study have never been studied in the past and literature lacks comparative analysis on weathering data for PSW. It should also be noted that the results estimated from this work show that the PSW blends result in lower *E_a_* values in comparison to virgin resin analysis shown in previous reports [25,26,52].

### 3.3. Analytical Solution Model

**Figure 9:**
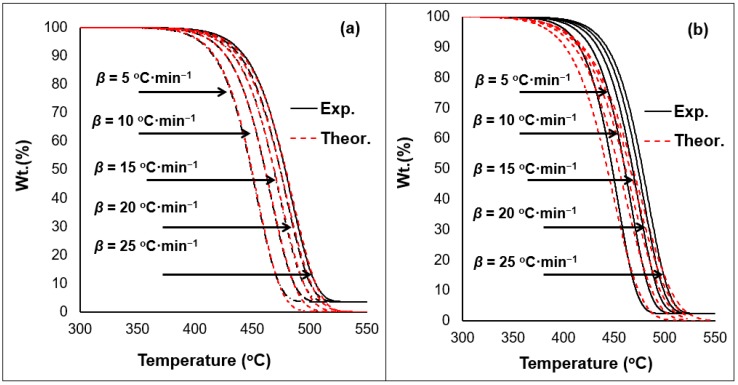
Experimental (Exp.) vs. theoretical (Theor.) results obtained from the analytical solution model for the (**a**) pre-exposed and (**b**) exposed 50/50 samples.

**Figure 10:**
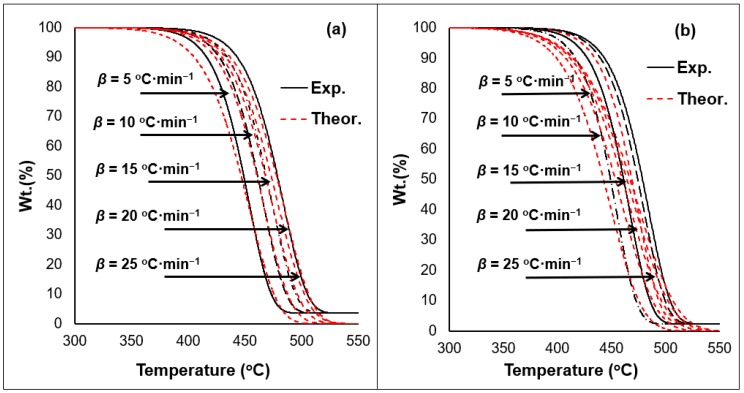
Experimental vs. theoretical results obtained from the analytical solution model for the (**a**) pre-exposed and (**b**) exposed 0/100 samples.

A mathematical formula was specifically designed to estimate the kinetic parameters of the materials under investigation. The analytical solution, previously depicted, has been used in the past to study polymeric blends [25,26,46]. Table 2 summarizes the results obtained by the model. The regression coefficients obtained for all investigated materials showed high correlations among the datasets (*r^2^* > 0.9). The model also enabled the development of theoretical results as the figures below show (Figure 9 and Figure 10).

The model established in this work was able to mimic the S-curve typically shown for PO polymers conversion. In addition, the ability to estimate the reaction order values representing the dataset of the TGA thermogram, shows the model’s superiority over other methods. Values estimated for the A_o_ fall within range to past works on PE polymers [25,26,52,53]. The solution has also confirmed that the exposure to weathering reduces the *E_a_* values (Table 2). The incorporation of waste increases the deviation at the onset temperature range of the materials tested. The higher the amount of waste in the blend, the more deviation is observed (Figure 9 and Figure 10). The overall regression coefficient of the model with respect to experimental values is within acceptable range (>0.9). However, to overcome this model limitation, the incorporation of the reaction model as a scalar rather than a parameter is suggested. This will reduce the dependency on additional parameters whilst maintaining the reaction order at a constant value (*n* = 1) which results in a linear solution capable of representing the case at hand with a logical assumption. Previous research has shown that majority of PO polymer will degrade with a reaction order equal close to or equal to unity [25,26,39,40,41,42,43,44,45]. Therefore, it is suggested to incorporate a scalar for the reaction order in the model to overcome the variation in the dataset resulting from the weathering exposure of the polymeric materials. The implementation of such mathematical frameworks has enabled the determination of the fact that plastic waste will result in lesser required amount of thermal energy to disintegrate after exposure to climatic conditions.

### 3.4. Implications in the Context of Plastic Pollution

As previously discussed, thermolysis can be of great aid in managing and valorizing PSW. These technologies have proven their viability in the past; in providing a viable solution to SW accumulation in the environment. It is also worth mentioning that various implications, touching base on sustainability and plastic waste pollution, fall within the framework of this study. The developed film blends originating from PSW, have proven their viability as a standalone produce comparable to ones on the market [33,34,54]. This also supports the claim that freshly discarded PSW with somewhat preserved integrity, allows the development of plastic film blends with acceptable market standards. The addition of PSW to virgin resin and having a recycling protocol that can valorize this particular type of waste, can be of immense importance in reducing environmental burdens. When such practices are promoted, it is anticipated that the associated carbon footprint of the plastic products conversion will reduce. This is due to the reduction on the dependency on petrochemical based processes associated with virgin resin production and conversion to plastic articles [55,56]. This can result in an industrial scale operation that can treat such type of SW. It can also aid in managing SW in a closed loop cycle in countries that depend solely on fossil fuels, such as Kuwait.

The study of the thermal properties of the materials considered in this work, have also revealed a number of interesting findings. The estimated degree of crystallinity for the virgin LLDPE samples was 25% based on the first heating cycle of the heat flow measurements [49]. This reveals the fact that the majority of the polymer’s structure belongs to the amorphous region of this semi-crystalline material. This makes the blends investigated susceptible to photo-oxidation as a consequence to oxygen permeability [47]. Nonetheless, the rearrangement of the molecular structure of the materials have not affected their applicability for degradation under enforced inert atmospheres. In fact, the reduction of the solid char after exposure to weathering leads to higher gas and liquid yields from pyrolysis. This can support the fact that such an approach is viable even when waste materials are used [57].

By investigating the materials thermograms, it can be noted that the blends exhibit high thermal stability. The onset temperature range (404 °C to 430 °C) is lower than those reported for virgin PE polymers [25,53]. This also makes recycling such blends quite advantageous when thermolysis technologies are employed. In addition, the estimated *E_a_* from the analytical solution model places its values between 195 kJ·min^−1^ to 235 kJ·min^−1^ (unexposed samples) and 145 kJ·min^−1^ to 175 kJ·min^−1^ (exposed samples). All of which point towards lesser energy requirements compared to PE polymers.

## 4. Conclusions

In order to rid the environment of plastic pollution and its associated burdens, accumulated plastic waste must be managed via optimal and cost-effective means. The goal of this work was to study the degradation reaction and associated kinetics parameters of various blends originating from reclaimed plastic solid waste (PSW). These blends were also exposed to accelerated weathering to the point of total degradation (e.g., disintegrated), in order to, simulate their exposure to real life climatic conditions. It was noted that the blends thermal stability were not affected by weathering. In fact, the waste blends showed lower thermal properties to those of similar virgin polymers. This makes such materials quite advantageous for treatment using thermolysis technologies, namely pyrolysis under inter atmospheres. The evolution of residual char was also detected to increase under such conditions, which makes the prospects for product recovery very high in comparison to virgin plastics. The estimated *E_a_* values from the model free method showed that weathering alters the required energy demand for such products treated under pyrolysis. In fact, the single step kinetics established by the model free methods indicated that the *E_a_* value is closely associated with the reaction order. It was also confirmed using a developed mathematical model that weathering reduces the *E_a_* values. This was attributed to the rearrangement of the molecular structure due to photo-degradation. It is recommended to study the evolution gases with respect to reaction time in such cases as a low-hanging fruit future plan. It is also recommended to start implementing strategies that consider thermolysis technologies as a means to treat reclaimed plastic waste to avoid future plastic pollution problems that many countries face.

## Figures and Tables

**Table 1:** Summary and Major Findings of Plastic Materials Weathering Studies Using the Means of Accelerated Aging.

Material Tested	Weathering/Testing Conditions	Main Findings	Reference
LDPE, LLDPE and HDPE	Weatherometer (WOM) exposure following ASTM G26 up to 1600 h. QUV exposure following ASTM G53 up to 800 h.	QUV^®^ conditions were more severe than WOM on polyolefin (PO) materials tested. The main chemical modifications were noted to be carbonyl formation of various types. Stability against oxidation were determined in the following order: HDPE > LLDPE > LDPE.	Gulmine et al. [27]
PP (un-stabilized) Recycled plastic using compression molding (7 min at 170 °C)	Elastocon heating cabinet (70 °C, air flow of 10 L·min^−1^) up to 8 days of exposure.	Hydroperoxides formed during aging are rapidly transformed into carbonyl groups upon compression molding.	Jansson et al. [28]
LLDPE (with and without hindered amine light stabilizers)	QUV following ASTM 4329 up to 50 days of continuous exposure.	Synergism of UV stabilizers and light transforming additives was noted to have an impact on stress at yield point up to 50%.	Al-Salem [29]
Wood/PP composites (WPC)	Xenon Test Chamber in accordance with ISO 4892-2 up to 1000 h of exposure.	*α-keto* carbonyl groups of cellulose increased together with vinyl groups due to PP oxidation after weathering	Turku and Karki [30]
Sago starch filled LLDPE composites	Dumbbell specimen in air oven at 70 °C exposed for 4 weeks.	Pro-oxidant presence manipulated carbonyl accumulation in comparison to the control samples.	Sharma et al. [31]
LLDPE/Waste Blends	QUV following ASTM 4329 up to 15 days of continuous exposure.	Waste content acts as a deteriorating agent in majority of blends due to weaker polymeric matrix.	Al-Salem et al. [12]

LDPE: low density polyethylene, LLDPE: linear low-density polyethylene, HDPE: high density polyethylene, ASTM: American Society for Testing and Materials (Method), PP: polypropylene.

**Table 2:** Apparent activation energy (*E_a_*), pre-exponential factor (A_o_), and reaction order (*n*) results determined using the developed analytical solution mathematical model.

Unexposed Samples				Exposed Samples		
*β* (°C·min^−1^)	A_o_ (min^−1^)	*E_a_* (kJ·min^−1^)	*n*	A_o_ (min^−1^)	*E_a_* (kJ·min^−1^)	*n*
Material Code: 50/50						
5	8.21 × 10^14^	235	1.1	4.93 × 10^10^	175	1.1
10	3.76 × 10^14^	230		2.65 × 10^10^	170	
15	1.16 × 10^14^	225		1.34 × 10^10^	165	
20	7.59 × 10^13^	220		6.65 × 10^9^	160	
25	3.24 × 10^13^	215		3.29 × 10^9^	155	
Material Code: 25/75						
5	6.06 × 10^13^	225	1.1	2.18 × 10^10^	170	1.1
10	4.26 × 10^13^	220		1.55 × 10^9^	165	
15	2.64 × 10^13^	215		6.14 × 10^9^	160	
20	1.55 × 10^13^	210		3.07 × 10^9^	155	
25	7.24 × 10^12^	205		1.52 × 10^9^	150	
**Material Code: 0/100**						
5	1.46 × 10^13^	215	1.1	9.74 × 10^9^	165	1.1
10	1.48 × 10^13^	210		5.49 × 10^9^	160	
15	6.87 × 10^12^	205		2.82 × 10^9^	155	
20	3.24 × 10^12^	200		1.42 × 10^9^	150	
25	1.41 × 10^12^	195		7.11 × 10^8^	145	

## Supplementary Materials

The following are available online at http://www.mdpi.com/1660-4601/16/3/395/s1; Figure S1: The various stages of the recycling procedure undertaken in this work showing (a) reclaimed plastic waste at landfill site, (b) storage of waste material, (c) transportation, (d) milling machine assembly, finally; (e) compounding of blends; Figure S2. Plate of (a) control specimens and (b) samples past the threshold limit in the accelerated weathering chamber (16 days). Source: Al-Salem [54]; Figure S3. Accelerated weathering procedure showing the (a) weathering chamber, (b) samples mounted on the exposure racks, (c) drying of samples and finally, (d) unloading of samples. Source: Al-Salem [56]; Table S1: Algebraic expressions for g(α) and f(α) indicating the most frequently used mechanism of solid state processes. Adapted from Al-Salem et al. [25,26]; Table S2: Degree of crystallinity (%) measured for the studied samples before and after exposure to threshold limit of accelerated weathering, Table S3: TG thermograms for the unexposed materials, Table S4: TG thermograms for the materials exposed to threshold limit.

## Abbreviations

A_o_	Pre-Exponential Factor (min^−1^)
BPA	Bisphenol-A
DEHP	Di-(2-ethylhexyl)phthalate
DSC	Differential Scanning Calorimeter
*E_a_*	Apparent Activation Energy (kJ·mol^−1^)
EU	European Union
f(α)	Reaction Model
FWO	Flynn-Wall-Ozawa Isoconversion Method
GDP	Gross Domestic Product
GHGs	Greenhouse Gases
HDPE	High Density Polyethylene
LDPE	Low Density Polyethylene
LLDPE	Linear Low Density Polyethylene
*m*	Mass of Polymeric Material at a Specific Time of Reaction
ME	Middle East
*m_f_*	Final Mass of Polymer
*m_o_*	Initial Mass of Polymer
MRF	Materials Recovery Facilities
MSW	Municipal Solid Waste
OECD	Organisation for Economic Co-operation and Development
PO	Polyolefin
PP	Polypropylene
PSW	Plastic Solid Waste
PVC	Polyvinyl Chloride
SW	Solid Waste
T	Reaction Temperature at Desired Time (K)
*t*	Reaction time (minutes)
TCT	Thermo-Chemical Treatment
TGA	Thermogravimetric Analysis
*T_m_*	Maximum Degradation Temperature (K)
UNGHS	United Nations Globally Harmonized System
*α*	Material Conversion
*β*	Heating Rate (°C·min^−1^)
