# Supplementary material for: On the Kinetics of Degradation Reaction Determined Post Accelerated Weathering of Polyolefin Plastic Waste Blends
Source: Int J Environ Res Public Health. 2019 Jan 30;16(3):395. doi: 10.3390/ijerph16030395 (PMC6388198; doi:10.3390/ijerph16030395)
Supplement: Supplementary file 1 [file ijerph-16-00395-s001.pdf]

Supplementary Materials File:

This file contains figures and images that support the work shown in the article, where it adds value to readers in various section and subsection.

List of Supplementary Figures:

Figure S1. The various stages of the recycling procedure undertaken in this work showing (a) reclaimed plastic waste at landfill site, (b) storage of waste material, (c) transportation, (d) milling machine assembly, finally; (e) compounding of blends,

Figure S2. Plate of (a) control specimens and (b) samples past the threshold limit in the accelerated weathering chamber (16 days),

Figure S3. Accelerated weathering procedure showing the (a) weathering chamber, (b) samples mounted on the exposure racks, (c) drying of samples and finally, (d) unloading of samples,

List of Supplementary Tables:

Table S1. Algebraic Expressions For g(α) and f(α) Indicating The Most Frequently Used Mechanism Of Solid State Processes,

Table S2. Degree of crystallinity (%) measured for the studied samples before and after exposure to threshold limit of accelerated weathering,

Table S3. TG Thermograms For the Unexposed Materials,

Table S4. TG Thermograms For the Materials Exposed to Threshold Limit.

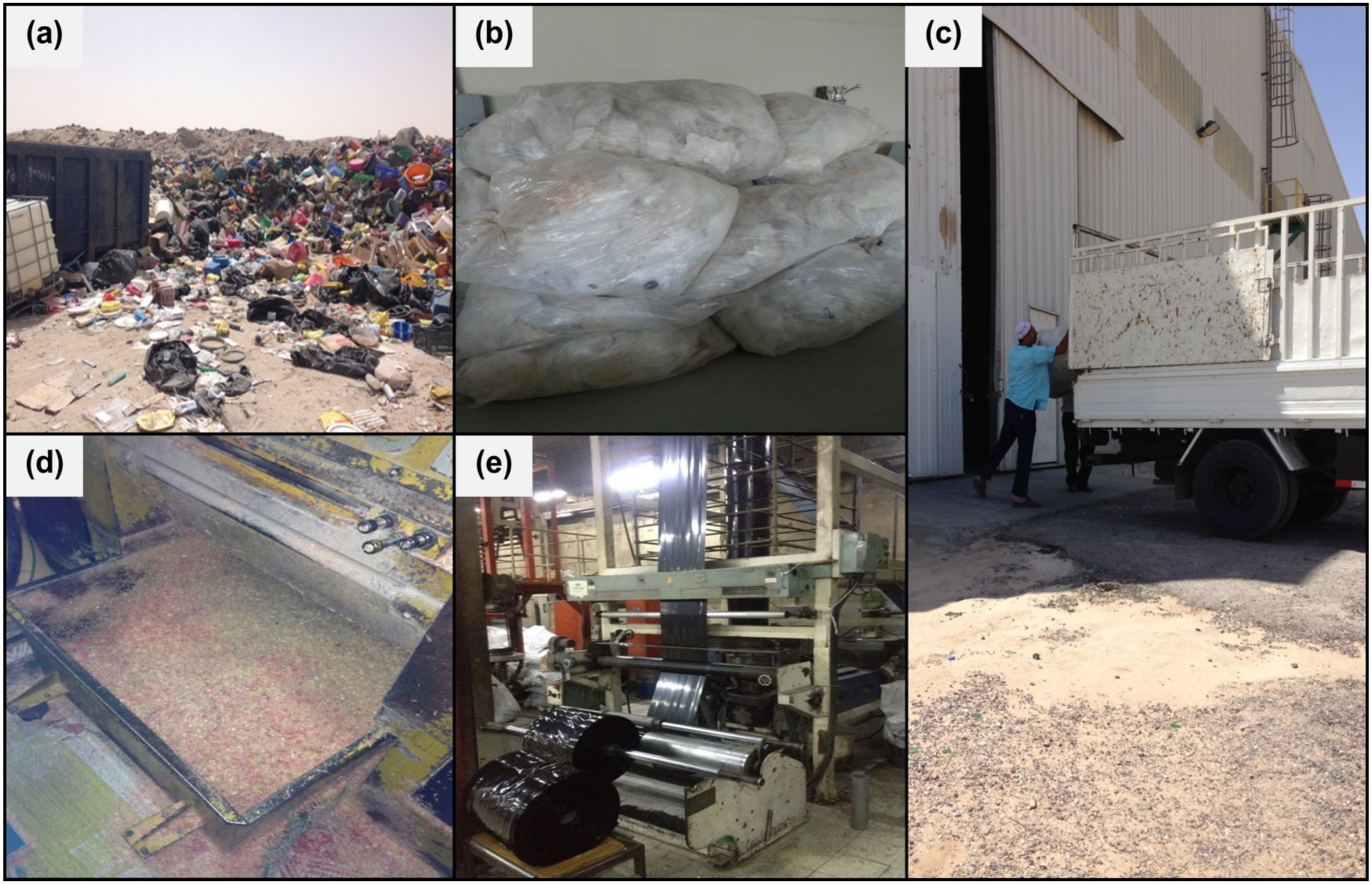

Figure S1. The various stages of the recycling procedure undertaken in this work showing (**a**) reclaimed plastic waste at landfill site, (**b**) storage of waste material, (**c**) transportation, (**d**) milling machine assembly, finally; (**e**) compounding of blends.

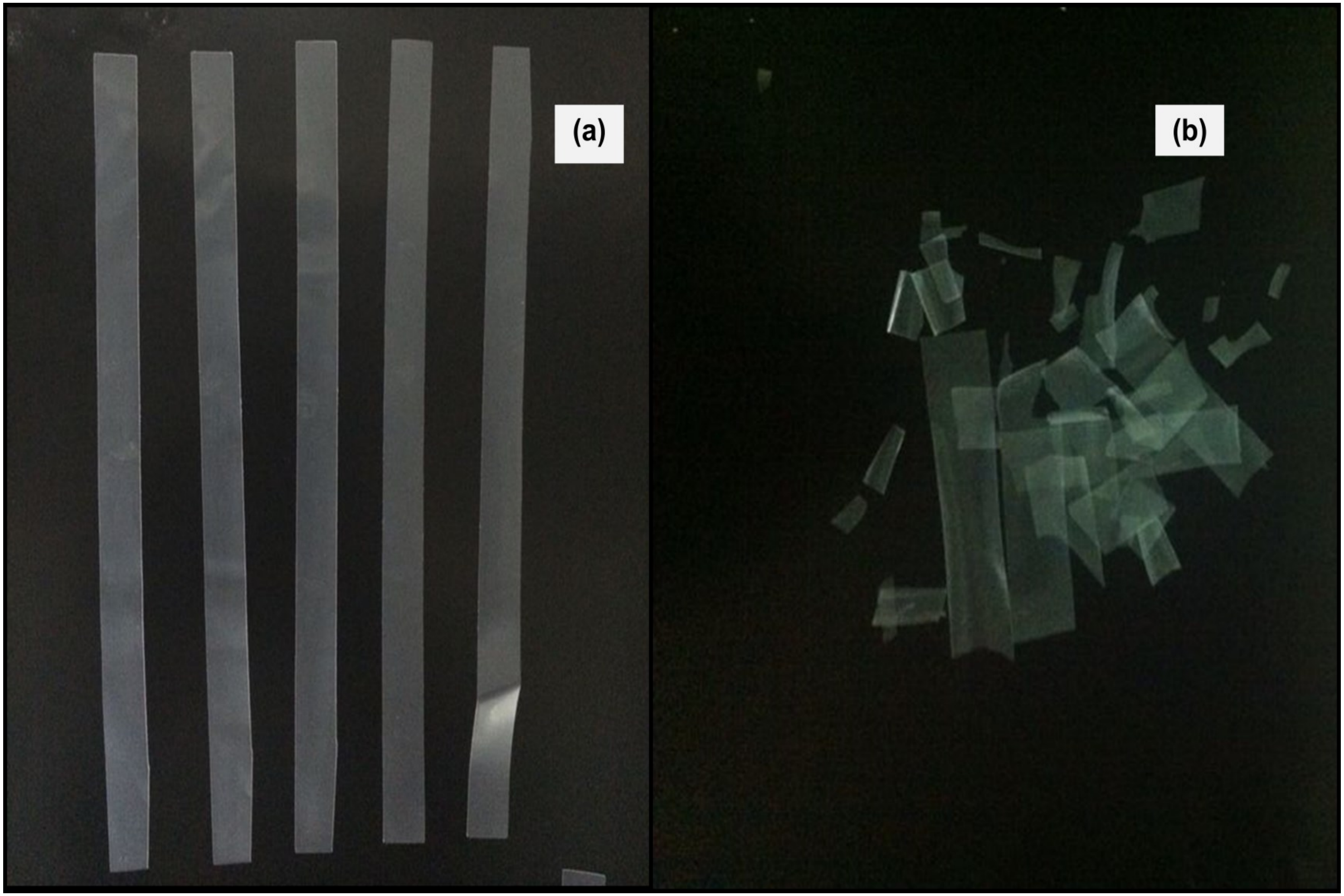

Figure S2. Plate of (**a**) control specimens and (**b**) samples past the threshold limit in the accelerated weathering chamber (16 days).

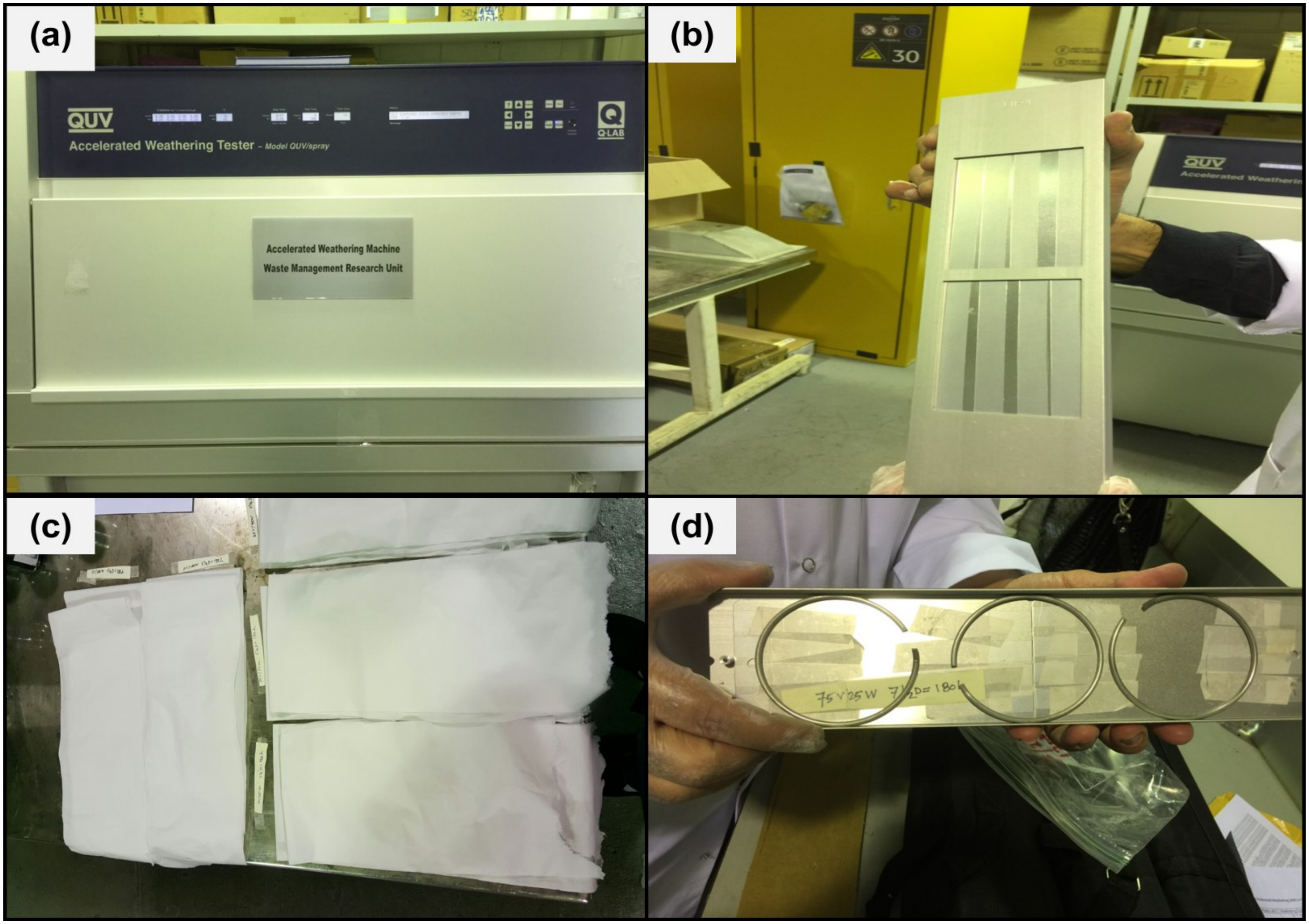

Figure S3. Accelerated weathering procedure showing the (**a**) weathering chamber, (**b**) samples mounted on the exposure racks, (**c**) drying of samples and finally, (**d**) unloading of samples.

Table S1. Algebraic Expressions For g(α) and f(α) Indicating The Most Frequently Used Mechanism Of Solid State Processes.

**Solid state Process Mechanism**	**f(α)**	**g(α)**
A2—Nucleation and growth (Avrami Eq.1)	$2(1-\alpha)[-\ln(1-\alpha)]^{1/2}$	$[-\ln((1-\alpha)]^{1/2}$
A3—Nucleation and growth (Avrami Eq.2)	$3(1-\alpha)[-\ln(1-\alpha)]^{2/3}$	$[-\ln((1-\alpha)]^{1/3}$
A4—Nucleation and growth (Avrami Eq.3)	$4(1-\alpha)[-\ln(1-\alpha)]^{3/4}$	$[-\ln((1-\alpha)]^{1/4}$
R2—Phase boundary controlled reaction (contracting area)	$2(1-\alpha)^{1/2}$	$[1-\ln((1-\alpha)]^{1/2}$
R3—Phase boundary controlled reaction (contracting volume)	$3(1-\alpha)^{2/3}$	$[1-\ln((1-\alpha)]^{1/3}$
D1—One dimensional diffusion	$1/2(\alpha)$	α^2
D2—Two dimensional diffusion (Valensi equation)	$[-\ln(1-\alpha)]^{-1}$	$(1-\alpha)\ln(1-\alpha)+\alpha$
D3—Three dimensional diffusion (Jander equation)	$(3/2)[1-(1-\alpha)^{1/3}]^{-1}(1-\alpha)^{2/3}$	$[1-(1-\alpha)^{1/3}]^2$
D4—Three dimensional diffusion (Ginstling-Brounshtein equation)	$(3/2)[1-(1-\alpha)^{1/3}]^{-1}$	$[1-(2/3)\alpha]-(1-\alpha)^{2/3}$
F1—Random nucleation with one nucleus on the individual particle	$1-\alpha$	$-\ln(1-\alpha)$
F2—Random nucleation with two nuclei on the individual particle	$(1-\alpha)^2$	$1/(1-\alpha)$
F3—Random nucleation with three nuclei on the individual particle	$(1/2)(1-\alpha)^3$	$1/(1-\alpha)^2$

Table S2. Degree of crystallinity (%) measured for the studied samples before and after exposure to threshold limit of accelerated weathering.

**Material Code: 50/50**	
**Exposure Condition**	**Crystallinity (%) ***
Unexposed	32
Exposed+	31
**Material Code: 25/75**	
**Exposure Condition**	**Crystallinity (%) ***
Unexposed	30
Exposed+	35
**Material Code: 0/100**	
**Exposure Condition**	**Crystallinity (%) ***
Unexposed	28
Exposed+	40

* Crystallinity (%) measured with reference to pure polyethylene samples between 60 °C to 130 °C on the first heat flow thermogram.

+ Exposure to threshold limit of samples was achieved after 11 days of continuous aging in the UV chamber.

Table 3. TG Thermograms For the Unexposed Materials.

Sample Formulation (Virgin/Waste) (wt%)	Heating Rate (°C·min^{-1})	Onset Temperature (°C)	Midset Temperature (°C)	Maximum Temperature (°C)	Inflection Point (°C)
50/50	5	404	448	512	453
	10	415	461	527	465
	15	422	468	536	472
	20	427	474	543	479
	25	430	478	546	483
25/75	5	404	448	512	452
	10	415	461	526	465
	15	422	468	535	472
	20	427	474	542	478
	25	430	478	547	484
0/100	5	404	448	512	452
	10	415	460	526	464
	15	422	468	535	472
	20	427	474	542	478
	25	430	478	547	484

Table S4. TG Thermograms For the Materials Exposed to Threshold Limit.

Sample Formulation (Virgin/Waste) (wt%)	Heating Rate (°C·min^{-1})	Onset Temperature (°C)	Midset Temperature (°C)	Maximum Temperature (°C)	Inflection Point (°C)
50/50	5	404	448	512	453
	10	414	460	526	465
	15	421	468	543	473
	20	426	473	549	479
	25	430	477	547	483
25/75	5	404	448	515	453
	10	414	460	529	465
	15	421	468	543	473
	20	426	473	549	479
	25	430	477	549	483
0/100	5	404	449	514	454
	10	415	461	528	466
	15	421	468	537	474
	20	426	473	544	482
	25	430	478	549	485
